# Selections and Abstracts

**Published:** 1900-07

**Authors:** 


### SELECTIONS AND ABSTRACTS.

---

### MODERN BUSINESS METHODS FOR THE MEDICAL PROFESSION.*

After studying bookkeeping, typewriting and shorthand, I served two years in a post-office under a postmaster who was very methodical. Everything had a place and was in its place. I then began to study medicine under two doctors, brothers. The contrast was extreme. Dirt everywhere, magazines and periodicals in great confusion, thousands of duplicate prescriptions filled several drawers, hundreds of letters packed in boxes, odds and ends anywhere and everywhere, second-hand furniture and cast-offs from their homes. I simply mention this to describe three out of every five offices in the land. I then undertook to get order out of chaos, and vowed that my office should never become so. I have since been engaged in three different offices, and have fitted up my office as described below. We all preach asepsis, and none of us make any pretense of heeding it in furnishing our offices, and few take advantage of modern plans for the business part of our work.

My office floor is covered with linoleum, which can be kept aseptic. Oiled or waxed floors are nice. Carpets are an abomination. I have adopted the Werner elastic bookcase system for my instruments and books. They are handy and very satisfactory. A roller-top desk is a necessity and well worth the money spent. Have in one pedestal several files to keep your letters in order, one drawer for the cards I shall describe, one drawer for stationery, another for examination blanks and blank reports. Label the pigeonholes, each for a distinct purpose, and you will have made an excellent start. Then when you leave your office close the desk; the drawers lock automatically, and no one can pry into your business. I have a shelf holding a large bottle of distilled water, an irrigator, glass jars containing bandages, gauze and cotton. An improved Harvard chair embellishes the room, and is really a necessity. A

---

* Read by W. A. Jolley, M.D., Rawlins, Wyo., before the Wyoming State Medical Society, Laramie, Wyo., October 10, 11, 1899.

screen hides the washstand, and is convenient to place before a window or door when desired.

A typewriter is a great convenience. Our speed in recording ideas is doubled, if not trebled, with a little practice, and in a few months, when we work the machine as automatically as a pen, we will be surprised to find how freely ideas will flow, and notice that our spelling, grammar and rhetoric have improved. The main objection to keeping notes is then done away with, for time is reduced to a minimum, legibility increased, and the matter is more compact. A full history can be placed on a 3 x 5 card. Prescriptions can be written legibly and accurately. Stop using lead pencils; use a fountain pen if you have no typewriter. I have these cards to keep records of my cases and their accounts. On one side I have a few blank spaces, which can be arranged according to need; on the other I have ledger ruling. I will have my next cards arranged with the name to be placed on the ledger side, as many of my cases need no record that cannot be placed in the ledger. If the record is too long I continue it on paper cut to the proper size. These I paste to the card. I attach letters from patients to their cards. Temperature charts are also pasted on.

I have a Physician's Visiting List, issued by the Maltine Company. I have used several of these, which are supplied gratis. From this I post to the cards. Cash accounts are marked with an x; credit accounts are marked Cr. At the end of a month I can easily ascertain amount of work done and cash collected, so do not use a cash-book. I have used ledgers, but find the cards more satisfactory: first cost is more, but in ten years' time it is not. It is never filled; every account is complete in itself, and not scattered through three or four ledgers. At the end of ten years our outfit is up to date. N. G. accounts and closed accounts are weeded out, and only active and open accounts are there. It is a labor-saver. No index is kept, for by means of guide cards each account can be found in an instant—no transferring from one ledger to another. In a lawsuit only the card involved will have to be sent to court. Cards can be carried in a pocket and can be used in a typewriter.

The bulk of modern medical literature is to be found in the magazines and papers, yet, on account of the abundance, it is inaccessible, for it would take hours to find all the articles on any desired subject in the stacks of papers that accumulate in every office. My magazines are kept on my desk until they are examined, then placed in order in my bookcase. At the end of a year I remove the advertising pages, pull out the cord or wire, arrange the papers in proper sequence, insert the index in the proper place, weight them down until solid, square the back edge and cover with glue. When dry I put on the covers of bristol board, which have been cut to the proper size, and with an awl punch three holes through cover and board as near the back edge as possible. From the back pass a cord through the middle hole, then through a side one, across the back to the other side hole, through it and then through the middle one again—this makes two cords in the middle one; place one on each side of the string running along the back and draw tight and tie, then pound the knot in the hole. With a suitable cloth cover the back edge and an inch of the cover; label as you wish. When solid take to a printing-office and have the edges trimmed, and you will have a handy book—not artistic, but pactical and satisfactory—at a cost of seven cents per book for material. Any printer will do it for 25 cents per volume. Then it will be a pleasure for you to refer to current literature. It is not very much harder to bind in the regular manner, but we need a few appliances and skill, while my method does not. I think to pay 75 cents to $1 to bind magazines is money thrown away.

All of us have been afflicted with the clipping habit, but in a short time we have scrap-books filled with such a mass of information that we are at sea when we wish to refer to it, even if we did keep an index. I have made a book out of large envelopes, binding them like magazines in layers of three, overlapping each other, then index them. I have seen advertisements of books made for the purpose. Several books can be made, each for a subject—surgery, medicine, medical societies, etc. In my text-books I make marginal references of any article which I think worthy.

I keep my bills in order in labeled envelopes, which I keep in a pigeon-hole in my desk. My labels read: coal, water, electric lights, papers, instruments, books, etc. After I have filed letters from patients with the cards, the bills, receipts, and society work, as described, few are left; these I file in letter cases.

It is disappointing to receive a letter from a prominent man written on poor paper and enclosed in a cheap envelope. I believe that we should use a good quality paper, with a neat card, then we will not be taken for a "cheap John" doctor. No thriving business firm uses poor stationery. Bill-heads should be neat and small. Our cards should be regulated according to use intended. A good printer can give the proper form.

I have covers made like those on my magazines, only not attached. In these I place my reprints, college, book and instrument catalogues. Duplicate prescriptions should be bound in books of 200; back edge labeled with numbers and date, and keep in compartments in desk which are made of the proper size. I do not keep mine, for I have the dates on my cards, and then I go to the drug store and see the original.

All of us have formulæ which we use repeatedly. Oh no! we write our prescriptions according to indications. You ask the druggist to see the files, and you will be surprised to find that empiricism had such a sway; for day after day appear the same prescriptions, and on some back shelf the druggist will have large bottles of Dr. X.'s cough medicine, liniment, salve, etc. We then will feel mortified to think that we have made so little use of our minds and the splendid list of drugs now in common use, but have prescribed M. N. & Co. Elix., etc. If you have a good druggist he can make an elegant preparation as is needed, if you will only cooperate. There are many preparations which we prescribe that we can make if we would only think and experiment. A firm in Denver makes a pleasant substitute for a poultice. I used a great quantity, then had a druggist make me a mixture of my own, which does as well.

I have several formulæ which the druggists know and keep prepared. In prescribing these I use the initials of the drug and the first two letters of my name. Cassjo means codeine, acetanilid, salol, and strychnine. In theory I think it would pay to keep our own drugs and employ a pharmacist, who could also aid us by doing the clerical work and assisting in operations.

I believe that it is the best policy to follow the methods of a practical business man. Make use of every new instrument which we think will help us. Also, utilize time- and labor-savers, such as telephones, electric lights, automobiles, etc. In fact, have the reputation of having the latest in everything, then a patient will rest satisfied that the very latest methods are being employed.

Advertising? Well, that is a subject that requires separate consideration. I have said nothing about collecting accounts, for each one seems to be a law unto itself.— *Western Med. Review.*

---

### HYPNOTIC ACTION OF APOMORPHINE WITHOUT NAUSEA.*

Apomorphine acts as a prompt and well-nigh infallible hypnotic, if injected subcutaneously in doses of about one-thirtieth of a grain. A careful search through standard text-books, and a large correspondence on this subject with representative medical men have demonstrated to me that the profession is ignorant of this important fact.

Although one-thirtieth of a grain is about the average hypnotic dose, yet for some patients this is too large, as it produces nausea, while in others a larger amount will cause no disagreeable symptoms. The dose should be so adjusted as to be large enough to produce sleep, and small enough to avoid nausea. This being only about one-third of the ordinary emetic dose, it is, of course, perfectly harmless.

I believe the use of apomorphine for the purpose of producing sleep without vomiting is original with me. The last edition of Potter's "Handbook of Materia Medica, Pharmacy and Therapeutics" (1899) contains on page 394 this sentence: "Apomorphine, though a derivative of morphine, is neither hypnotic nor narcotic in any degree."

Such a statement in so recent and authoritative a work as this shows that the remarkable properties of this drug are not generally known to the medical profession.

When thus administered, apomorphine acts as a hypnotic with

*Prize Paper in Literary contest of *Merck's Archives*, by Charles J. Douglas, M.D., Physician in charge of the Walter Baker Sanitarium, Boston, Mass.

precision. Both in mild insomnia and in furious delirium I find that it produces sound sleep in from five to twenty-five minutes. On waking, the patient feels none of the unpleasant symptoms that usually follow sleep induced by drugs. The sleep of this remedy is refreshing and restful. As its action is so prompt, it is advisable usually to administer it when the patient is in bed, or quite ready for bed. If a delirious patient refuses to go to bed, this hypnotic will cause him to voluntarily lie down in a few minutes, and sleep will follow. Its direct hypnotic action appears to last from one to two hours, but in many cases the patient will sleep all night, if promptly put by sleep on this remedy on going to bed. If more is needed in extreme cases to produce an eight-hour sleep, a small dose of some slower acting hypnotic will aid in keeping the patient asleep that length of time.

There is no possibility of a "drug habit" being formed, as it becomes a vigorous emetic if the dose be increased. There are no cumulative effects from this remedy, hence it may be given at frequent intervals without harm. These small hypnotic doses usually accelerate the heart's action slightly.

I discovered accidentally that apomorphine becomes inert if dissolved in a saturated solution of boracic acid. I made the mixture for the purpose of securing the antiseptic action of the latter drug, and found that I had completely neutralized the remedy, both as a hypnotic and as an emetic. I have failed to find in medical literature any reference to the antagonism between these two drugs.

During the last four years I have given apomorphine in private and sanitarium practice to three hundred patients, I think, and during that time I have found two or three individuals on whom it had but slight hypnotic or emetic effect. One in a hundred is perhaps a fair estimate.

In such cases emesis cannot be produced usually, even when the largest doses are given.

Another idiosyncrasy that I have occasionally observed is a peculiar susceptibility to the emetic action of this drug.

CASE 1.—A man forty-five years of age. He had had no sleep of any consequence for over two weeks, and had used many kinds of narcotics without obtaining relief. The first night I saw him I

administered hypodermically one-thirtieth of a grain of apomorphine at bedtime. In about twenty minutes he was asleep and slept till morning, greatly to his astonishment. After securing a few nights of refreshing sleep in this way he was soon able to sleep without aid.

CASE 2.—Dr. F. had been under my care at the sanitarium for several weeks as a morphine patient. After the complete withdrawal of that drug he became the victim of persistent insomnia, which no ordinary dose of the usual hypnotics would relieve. The injection of apomorphine never failed to put him to sleep in a few minutes without nausea. He reported that the sleep thus induced was always refreshing and seemed natural—quite unlike the sleep of other narcotics.

CASE 3.—An epilectic with violent and destructive mania following every attack. Neither morphine, bromide nor chloral would materially modify the delirium or produce sleep. I finally resorted to apomorphine, which invariably puts him to sleep in ten or fifteen minutes, the sleep lasting from one to four hours. On waking, he is always greatly improved and usually free from delirium.

CASE 4.—A strong mechanic forty-eight years of age. Delirium tremens in the fighting stage. I injected one-thirtieth of a grain of apomorphine, and he went to sleep in ten minutes without vomiting and slept about an hour. When he awoke the delirium was gone.

The advantages of apomorphine as a hypnotic may be summarized somewhat as follows:

1. Safety.

2. Promptness—producing sleep in less than half an hour, and frequently in ten minutes.

3. Certainty of action in most cases, even in the wildest delirium.

4. Refreshing and natural character of the sleep.

5. No danger of habit.

In addition to its hypnotic action, it is also a cardiac stimulant, sudorific and antispasmodic. Further experiments may show that it is also antipyretic and analgesic. Dr. Tilton has called my attention to the interesting fact that when used as an emetic in croup it is followed by a decline in the fever. Possibly the small hypnotic dose will also produce this antipyretic effect in other fevers.

It is to be hoped that many physicians will at once begin the investigation of this remedy along the lines I have indicated, and that they will give their clinical experience to the profession through our medical journals. Such a course will demonstrate, I believe, that in apomorphine we have, unsuspectingly, been carrying about with us a drug possessing remarkable properties and a wide range of usefulness.

---

### PLUGGING OF THE UTERUS IN ABORTION.

Dr. J. Keogh Murphy (*Treatment*, December 28th, 1899) in a thesis for the M. D. degree of the University of Cambridge, says:

"I would venture to put forward what I consider to be safe rules for the employment of plugging: 1. Plugging may be considered to be good treatment—indeed, the best—in cases of inevitable abortion, where the hemorrhage is very serious and the os contracted so that neither a finger nor the curette can be passed into the uterine cavity. 2. In such cases plugging should only be used as a means of stimulating uterine contractions so as to obtain sufficient dilatation of the cervix to be able to empty the uterus. 3. Under no circumstances should plugging be used in incomplete abortion.

"Plugging, indeed, in cases of abortion only finds its place in the first three months of pregnancy; here the hemorrhage may be very alarming before there is any dilatation of the os—indeed, I consider the earlier the pregnancy, the graver the hemorrhage is likely to be. After the third month the hemorrhage is not likely to be severe before the os is sufficiently dilated to allow the finger to be introduced.

"I consider, even in the early months of pregnancy, the cases where plugging is really needed are very rare indeed. To illustrate from my own cases: out of nearly four hundred cases I only had to plug the vagina three times, and in one of these it was quite unnecessary; but I had no curette with me at the time, and the woman had lost a very great quantity of blood. Most certainly, when used, plugging *was* effectual; it completely controlled the hemorrhage, and after the plug was removed the os was found to be sufficiently dilated.

"The advantages of plugging, to my mind, are: 1. That it is not a safe operation unless done with great cleanliness, and aseptic materials, the great pressure around the cervix causing ready absorption of septic material. 2. If it is to be effectual, it is a very painful operation, necessitating an anesthetic. 3. It is a temporary, not a final, measure, requiring to be repeated perhaps indefinitely.

"To shortly enumerate the routine of the operation itself which should be followed: A vaginal douche should always be given, the vulva being first cleansed with soap and water. The bladder and rectum should be always emptied before plugging. A speculum is really never needed, two fingers of one hand pressing down the perineum being much better, a speculum giving rise to difficulty in withdrawal and loosening of the plug. If possible, plug round the cervix with gauze, but for the vagina I would strongly recommend small balls of absorbent wool wrung out in an antiseptic, preferably lysol; the kite-tail of lint soaked in oil and fastened together by strings, recommended by some authors—a wonderful and fearsome device—should be eschewed.

"The plug should always be removed in twelve hours at the longest, and the vagina douched again after removal, care being taken to see that no plug is left behind in the fornices. For this reason I advise a single piece of gauze round the cervix. In such cases the use of the catheter will seldom be required owing to the preceding loss of blood. If plugging be done at all, it should be thoroughly done; it is not easy to err on the score of tightness, short of actual violence. The necessarily large consumption of cotton wool should not be feared, remembering that a plug to be of any use at all must not only fill but distend the vagina and press up the uterus."

The author then recommends boldly emptying the uterus by the finger or by the curette as soon as the cervix is sufficiently dilated to do so. This he believes to be the correct treatment in all cases, and he would even recommend it to be done in all cases where hemorrhage, even though slight, continues, for here we may be certain that the abortion is incomplete.

The author submits the following conclusions: 1. Plugging in early abortion is rarely necessary, and when employed should be used only with the definite hope of dilating the cervix. 2. Ergot and other drugs should not be used unless the case is complete, and they will then be unnecessary. 3. In about five per cent. of all cases (twenty to four hundred) operative removal of the ovum must be undertaken. He would advocate the early and thorough treatment of inevitable and incomplete abortions, as well as the entire safety of the treatment.—*N. Y. Med. Jour.*

---

### SO-CALLED ESSENTIAL HEMORRHAGE FROM THE KIDNEYS.

From time to time cases are encountered of renal hematuria in which no demonstrable lesion exists. We have recently had the pleasure of reading a memoir by Dr. Raphael Robinson, of Paris, who has devoted himself to the study of this interesting and obscure question.*

Hemorrhages of this nature occurring without obvious cause, are met with in young as well as in aged subjects. They take place suddenly, often in consequence of fatigue, and the quantity of blood passed varies within wide limits. The color is of a deep red or almost blackish, and upon examination under the microscope red blood-corpuscles, more or less altered, are perceived, but, in general, no renal element is present. In some cases there is more or less pain, but no fever. Hemostatics, far from being effectual, serve only to prolong the flow and occasion gastric disturbance. Spontaneous arrest of the hemorrhage often occurs.

It is known that certain poisons and certain toxins cause hemorrhage in general and hematuria in particular. On the other hand,

*Pathogénie et Traitement des Hematuries renales dites essentielles. Mémoire accompagné d'une Lettre de M. le Dr. Albarran, Professeur agrégé a la Faculté de Médecine, Chirurgien des Hôpitaux de Paris. Par Dr. Raphaël Robinson. Paris: Jouve & Boyer, 1899.

many persons, though healthy in appearance, are troubled by continuous nutritional disorders which may at length produce sclerosis, friability of blood-vessels, etc.

Dr. Robinson next proceeds to describe several cases which he had observed in private practice. In one patient hæmaturia came on in consequence of fatigue, lasted three days, and was succeeded by abundant epistaxis and purpuric spots. When these latter disappeared the hematuria recurred, but after a month ceased, and the patient has ever since—a period of seven years—been perfectly well. In a second case hematuria was accompanied by severe pain in the kidneys. Purpura was also present. The third patient belonged to a family of bleeders. He suffered from incoercible epistaxis, hemorrhage from the bowel, the lung and kidney. The hemorrhages came and went spontaneously, uninfluenced by medication. The man dying of a brain-trouble, the cerebral vessels were found enormously dilated. According to the classical descriptions, the first of these foregoing cases is a type of purpura, the second of scorbutus, and the third of hemophilia.

Essential hematuria has been divided into six classes: that dependent upon nephroptosis; that attended with neuralgia; that associated with hemophilia, with purpura, with albuminuria; and pure essential hematuria. The first of these classes is due to mechanical alterations; hematuria with albuminuria is connected with nephritis, for there are albumin and casts in the urine. The presence of neuralgia raises the suspicion of gravel or nephritis. In hemophilia and purpura the hematuria coexists with other hemorrhages.

In essential hæmaturia no renal lesion is found. In this connection two cases are cited by the author. The first was that of a man who had had rickets in his childhood. After a long walk he began to pass blood in the urine. The general condition was normal and there was no local pain. A year later there was a second attack of hematuria. The second patient was a neurotic and rachitic youth. He lived in a marshy district, but had never suffered from intermittent fever. In both these individuals the nutritive conditions were below normal from childhood. Rachitics eliminate comparatively enormous quantities of the salts and the chlorides, and oxidation is comparatively retarded. The bony lesions are the result of this decalcification. The blood also becomes poor in lime-salts and loses its coagulability. The kidney is overtasked in the work of removing the salts, and thus various factors combine to occasion renal hematuria. Coexistent hemorrhages are witnessed in acute rachitis, or Barlow's disease. Dr. Johannes Seitz, of Zurich, recently reported a case in which hematuria accompanied hemorrhage from the periosteum in a babe of ten months. Nothing is found in the urine of such patients to indicate the presence of nephritis, but a number of varieties of micro-organisms are observed. Our author, therefore, concludes that the toxins produced by these bacteria may cause hemorrhage from an organ particularly exposed, the kidney in this case.

That which happens in rachitis may equally occur in the victim of rheumatism, gout, hereditary syphilis, diabetes, etc. For all these maladies bear heavily upon the kidney, which is liable to succumb to an attack of a bacterial poison. The case of a rachitic child, four years of age, is then cited. The patient dwelt in a marshy district, but had never been ill. He was taken to a city in which scarlatina prevailed. There had been no case in the family or neighborhood. Suddenly one day the boy passed black urine. There seemed to be nothing adequate to explain the fact. There was no fever or sore throat. Yet, upon close examination slight desquamation was detected. After several days the signs of a mild endocarditis were recognized. Trousseau has mentioned such cases, and Professor Dieulafoy has witnessed hematuria as a consequence of infectious angina. Dr. Robinson next alludes to hematuria occurring during pregnancy, and quotes several cases in which this manifestation occurred in the absence of renal complication. He gives reasons for believing that such hemorrhages are due to alterations in the composition of the blood. They have also been known to take place in typhoid fever. The prognosis of essential hematuria is generally favorable, and Dr. Robinson has never met with a fatal case.—*Med. Bulletin.*

---

**This Journal and Atlanta Semi-weekly Journal one year for $1.00 cash in advance. No checks. Strictly to new subscribers.**

### A FRENCH SURGEON OF THE OLD SCHOOL.

At the annual meeting of the Société de Chirurgie, held a few weeks ago, M. Paul Reclus gave an interesting account of the career of Maisonneuve, whose name is already moss-grown, as if he had been half a century in his grave, though he died only three years ago. Maisonneuve was a pioneer in many previously unexplored regions of surgery, says the *British Medical Journal*, and he had the fate which too often befalls the man who is in advance of his time. He opened up more than one path which has since led others to lands flowing with milk and honey, but which brought him only to a Dead Sea of failure and disappointment. Not that he would have acknowledged defeat; he was ever a fighter, and to him doubtless the joy of the fray was in itself worth living for. But a spirit so bold and a genius so inventive might have accomplished far more than it did had it been his fate to work not in the dimness of the misty dawn of scientific surgery, but in the fuller light of a later day. Maisonneuve was born of good middle-class stock at Nantes in 1809. He had a brilliant career as a student, the teachers who influenced him most being Dupuytren and Récamier.

As a hospital surgeon he soon distinguished himself by originality in conception and boldness in execution. In 1845 he proved by experiments on animals the feasibility of intestinal anastomosis, but he was thought by the surgeons of that day to have imagined a vain thing. He was the first to perform blepharorrhaphy, to use forcible dilatation in stricture of the rectum, to tie the vertebral artery. He did excellent work on fracture of the fibula, on hip disease, on hernia. But his name will probably be remembered chiefly in association with internal urethrotomy.

Maisonneuve was too strong a man not to have enemies, and his manners had not that repose which suits the inhabitants of academic Olympus. He is reported to have said to a distinguished surgeon who was lamenting the bad condition of his hospital as conducing to a high rate of mortality, "There are no bad hospitals; there are only bad surgeons." To a student new to Paris, who asked for guidance as to the choice of a surgical teacher, he said,

"There are but two surgeons in Paris, Chassaignac and myself; and Chassaignac, too, is an imbecile." Tumors that no one else would touch were sent to Maisonneuve, who dared all that might become a surgeon, and perhaps a little more. So free was he with the knife that a house-surgeon is said to have asked on one occasion which part of the patient was to be carried back to bed. He was like Othello, rude in his speech, and he was something of a bully to his pupils. But he sometimes found his match. A student, who was afterwards one of the leading physicians in Paris, attracted Maisonneuve's notice by the foppishness of his dress and a certain affectation in his speech. The surgeon said to him, with a snarl of contempt, "Well, young gentleman, do you know what distinguishes a man from an ape?" The student coolly answered, "Yes, sir; politeness." The bystanders expected to see the presumptuous youth crushed by the wrath of the great man; but Maisonneuve only laughed, asked the lad to dinner, and was ever afterwards one of his staunchest friends.

In 1872 came the time for retirement from public work, and after 1879 Paris knew Maisonneuve no more. He buried himself in a country house in Brittany. He made no acquaintances among his neighbors, and received no visitors. But from miles around there came poor people on foot, on horse or donkey back, in carts and in wheelbarrows, to whom he freely gave the benefit of his skill, dressing their sores, setting their broken bones, breaking down ankyloses, reducing hernias, and removing cataracts. When he died the country folk crowded around his coffin for three days to sprinkle holy water on his dead body and pray for the repose of his soul; and to this day they bless the memory of the old man with the rough tongue but the skilful hand which wrought miracles of cure.

---

### SOME SURGICAL ASPECTS OF VOMITING.

The *Brit. Med. Jour.* for March 26th contains a paper by Mr. William H. Bennett, which calls for some comment. After pointing that in some instances the broncho-pneumonia which follows the administration of an anesthetic is due to the entry of vomited matter into the lungs, he rightly insists that the stomach should be empty before producing anesthesia, and this is all the more imperative in cases of fecal vomiting, because, apart from the risk of the vomited matter entering the lungs, it is in the highest degree desirable that any poisonous fecal matter in the stomach should be washed out.

But when Mr. Bennett attempts to differentiate between fecal vomiting that will get well without operation by "the casting off of the complaint" in the vomited matter, and cases of fecal vomiting that will end fatally if not operated on, we do not hesitate to say that he is unintentionally doing grievous harm by lending his name to such pernicious doctrine. Several cases are quoted in which recovery without operation took place after large quantities of fecal matter had been vomited, and in each of these Mr. Bennett noted that there was absence of progressive distention of the abdomen. Reasoning from these and probably from other unrecorded cases, he is of opinion "that in abdominal disease or injury, feculent vomiting, even when persistent, is no positive indication for surgical interference unless it is accompanied by increasing abdominal distention."

No one will doubt the accuracy of Mr. Bennett's observation, nor the significance of progressive abdominal distention, but are we to wait in every case of persistent fecal vomiting until it is evident that the abdomen is becoming extended?

For years, in season and out of season, surgeons have been insisting on the importance of early operation in cases of intestinal obstruction before the system becomes poisoned by the horribly offensive and highly toxic intestinal contents, and with emphasis we would add before the intestinal muscular tissue has become paralyzed and progressive distention of the abdomen becomes possible. Whatever value lies in Mr. Bennett's observation, it can only be applicable to abdominal cases, such as those of incarcerated feces, or impacted gall-stones, where the diagnosis does not preclude a cure by natural means; and we prefer to think that Mr. Bennett had these cases, and not "all cases abdominal disease and injury" in his mind at the time he wrote his article; and we hope he will seek an early opportunity of amending a statement which, coming from so distinguished and well-known a source, cannot fail to do much mischief.

NOTE.—The above commments by Mr. Priestly Leech in the *Quarterly Med. Jour.* are, in our opinion, so appropriate that we reprint them without further comment.

---

### HOW TO LIVE A CENTURY.

First, live as much as possible out of doors, never letting a day pass without spending at least three or four hours in the open air.

Second, keep all the powers of mind and body occupied in congenial work. The muscles should be developed and the mind kept active.

Third, avoid excesses of all kinds, whether of food, drink or of whatever nature they may be. Be moderate in all things.

Fourth, never despair. Be cheerful at all times. Never give way to anger. Never let the trials of one day pass over to the next.

The period from fifty to seventy-five should not be passed in idleness or abandonent of all work. Here is where a great many men fall. They resign all care of interest in worldly affairs, and rest of body and mind begins. They throw up their business and retire to private life, which in too many cases proves to be a suicidal policy.

During the next period—the period from seventy-five to one hundred years, while the powers of life are at their lowest ebb—one cannot be too careful about catching cold. Bronchitis is a most prolific cause of death in the aged. During this last period rest should be in abundance.

Anybody who can follow these directions ought to live to be one hundred years old at least.—*Exchange.*

---

***NOTICE.—This JOURNAL and THE INTERNATIONAL JOURNAL OF SURGERY one year for $1.00 cash, to new subscribers. Out-of-town checks not accepted unless 10c. is added for cost of collection. Strictly to new subscribers.***

### WILLIAM HARVEY.*

Those of us that have enjoyed the privilege of securing a medical education during the last decade of the nineteenth century have been instructed in certain facts that are accepted as such and concerning which there is no question. It is well for us occasionally to cast a retrospective glance over the ground that has been traversed by our predecessors and to try to realize how the paths that we follow so confidently at the present day have been blazed through the dense forests of prejudice, habit, and obstruction.

One of the truths that is taught to the pupil who takes his first lessons in anatomy and physiology in the common schools is that the blood, the tissue for the conveyance of nourishment and of oxygen to all the cells in the economy, passes from a central organ, the heart, through a system of arteries and capillaries, to the tissues; thence through the veins back to the heart, laden with effete matter; that it is then sent through the arteries and the capillaries of the lungs to be purified; and that it is then retured to the heart, from which it again starts on its journey of alimentation.

The circulation of the blood was not understood or admitted by the pioneers in medical study, and the secret of the passage of the blood from the heart to the tissues and from the tissues to the heart lay hidden until, in 1628, William Harvey announced it to the scientific world.

It will be advantageous for us to spend a short time in a study of the principal events in the life of this man, of the accepted teaching concerning the blood and the blood-paths at the time his work appeared, of his methods of work, and of the reception accorded to his demonstration.

William Harvey was born April 1, 1578, at Folkestone, Kent, England. His parents were in easy circumstances and were able to give their son an education that was of the best. At the age of ten years he entered the grammar school at Canterbury. At sixteen he was able to begin the course at Cains-Gonvil College, Cambridge, from which he graduated in 1597, at the age of nineteen years, with the degree of Bachelor of Arts. In 1598 he entered

* Read by John M. Swan, M.D., of Philadelphia, before the John Guiteras Medical Society, University of Pennsylvania, February 5, 1900.

the University of Padua, where he devoted five years to the acquirement of his professional education. In 1602 this institution gave Harvey the degree of "doctor of physic with license to practice and to teach arts and medicine in every land and seat of learning." It is interesting to note that at that day the candidates for the degree of "doctor of physic" were not younger than are the majority of the students who receive their diplomas at the present time. It was not, however, until the age of twenty-six that Harvey began to practice medicine in London. He was elected to the post of physician of St. Bartholomew's Hospital five years after beginning his practice. At the age of thirty-seven he was chosen to deliver the Caldwal lectures on anatomy and surgery at the College of Physicians. He was appointed one of the physicians to James I., and was attached to the royal retinue for many years following the fortunes, or possibly the misfortunes, of King Charles I. through the stormy period of the Civil War, but always as a physician, never as a politician. It is said that during the battle of Edge-Hill Harvey retired behind a hedge with the Prince and the Duke of York, with whose care he was charged, and employed his time in reading. He resigned from Charles's service at the age of sixty-eight.

The work on the circulation of the blood, "Exercitatio Anatomica de Motu Cordis et Sanguinis" appeared in 1628. An important work on Animal Generation, "De Generatione Animalium," appeared in 1651, and in the following year Harvey was looked up to as the most celebrated anatomist and physician of the age, and the College of Physician unveiled a statue of him with appropriate ceremonies. He died June 3, 1657, at the age of 79 years, from the effects of gout, which produced a cerebral hemorrhage, first manifested by "dead palsy" in the tongue.

At the period during which William Harvey was pursuing his medical studies, the anatomy and the physiology of the human body was little understood and the knowledge of the subject was helplessly befogged by crude theories and by speculations that seem to us, at this time, absurd. Galen was then the anatomic and physiologic authority, and, although he had some ideas that, when interpreted in the light of present knowledge, have led some modern students to think that the circluation of the blood was known to him, the bulk of his writings show that he and his contemporaries were far from appreciating the circulation as we know it. The heart, the pulmonary artery, and the pulmonary veins, the aorta and its branches, and the venæ cavæ and their tributaries were known to Galen. But while it was admitted that the right ventricle contained blood, the left ventriele was thought to contain spirits. The pulmonary artery was supposed to carry blood to the lungs for their nourishment, but the pulmonary vein was believed to carry spirits from the lung to the heart. The heart was considered to be the source of heat, and was placed between the lungs so that the latter organs might fan and refrigerate the former. The systole and the diastole of the heart were understood, but the diastole was believed to be the important act. It was taught by Galen that during diastole air was sucked through the pulmonary vein into the left ventricle, and a small amount of blood passed through the pores in the interventricular septum to the left ventricle, where it mixed with the spirits therein contained. The systole served to send the "fuliginous vapors" to the lung mixed with a little spirituous blood for the nourishment of those organs. The arteries, too, were described as possessing diastole and systole: during diastole they drew spirituous blood from the left ventricle and during systole they emitted "fuliginous vapors" through the pores of the flesh and the skin. The liver was believed to be the laboratory of the blood and the source of the nutrient blood for the body. Anastomoses were mentioned, but were not understood by the anatomists of the time because they failed utterly to grasp the idea of the heart as the motive power of the blood-current.

Such were the accepted teachings concerning the movement of the blood in 1628. In that year, after much careful observation, Harvey published his work on the motion of the heart and the blood. For nine years he had taught to his classes the theory that he now placed before the critics of his time for their verdict. For nine years he had demonstrated to his colleagues and to his students the facts that he set forth in his treatise, proving them by many dissections and by convincing arguments. He had made dissections of the human body and dissections of both living and dead animals for the purpose of illustrating the great truth that his genius and painstaking research had brought to light. So confident was he of the value of comparative anatomy in the clearing up of obscure points in the human anatomy that he is led to make the following statement: "Had anatomists only been as conversant with the dissection of the lower animals as they are with that of the human body, the matters that have hitherto kept them in a perplexity of doubt would, in my opinion, have met them freed from every kind of difficulty."

One of the experiments that Harvey used to demonstrate the fact that the blood did not pass from the right ventricle to the left ventricle through the pores in the interventricular septum is of interest. He obtained the body of a man who had been recently hung. Having tied the pulmonary artery, the pulmonary veins, and the aorta and incised the left ventricle, he passed a tube through the vena cava into the right ventricle and forcibly injected water into the right side of the heart, which became much distended. No blood or water came into the left ventricle, however. On the other hand, when the ligatures were removed and water was injected into the pulmonary artery, both blood and water appeared through the opening in the left ventricle.

In the dedication of his work to his colleagues of the Royal College of Physicians, he modestly proclaims his reasons for advancing his views for general adoption. "My dear Colleagues, I had no purpose to swell this treatise into a large volume by quoting the names and writings of anatomists, or to make a parade of the strength of my memory, the extent of my reading, and the amount of my pains; because I profess both to learn and to teach anatomy, not from books but from dissections; not from the positions of philosophers but from the fabric of nature; and then because I do not think it right or proper to strive to take from the ancients any honor that is their due, nor yet to dispute with the moderns, and enter into controversy with those who have excelled in anatomy and been my teachers."

His work was not complete: year by year men, working on the same lines of careful dissection, conscientious experiment, earnest thought, and modest advancemeut of new ideas have broadened our views of the subject of which Harvey laid the foundation. His views are concisely summarized in chapter xvi. of the thesis under consideration.

"Conclusion of the Demonstration of the Circulation.

"And now I may be allowed to give in brief my views of the circulation of the blood and to propose it for general adoption.

"Since all things, both arguments and ocular demonstration show that the blood passes through the lungs and heart by the action of the ventricles and is sent for distribution to all parts of the body, where it makes its way into the veins and the pores of the flesh, and then flows by the veins from the circumference on every side to the center, from the lesser to the greater veins, and is by them finally discharged into the vena cava and right auricle of the heart, and this in such a quantity or in such a flux and reflux thither by the arteries, hither by the veins, as cannot possibly be supplied by the ingesta, and is much greater than can be required for mere purposes of nutrition; it is absolutely necessary to conclude that the blood in the animal body is impelled in a circle and is in a state of ceaseless motion; that this is the act or function which the heart performs by means of its pulse; and that it is the sole and only end of the motion and contraction of the heart."

The work was received with a storm of protest from the anatomists of the day and many and violent were the attacks made on the new theory. These attacks were chiefly made, however, by the older men; the younger men of the profession, those who were open to conviction and who were not saturated with prejudice in favor of the Galenic theories, accepted the demonstrated truth and taught it to others.

Although the criticisms were hostile to a degree and, in some instances descended into personal abuse, Harvey, who never approved of heated personal discussions replied to but one, John Riolanus, who was Regius Professor of Anatomy and Botany in the University of Paris. He accepted the theory of the circulation of the blood partly, but not entirely. He says that "the blood contained in the portal vein does not circulate like that in the vena cava. There is some blood that circulates and the circulatory vessels are the aorta and the vena cava, but the continuations of these trunks have no circulation because the blood is effused into all the parts of the second and third regions where it remains for the purpose of nutrition; nor does it return to any greater vessels unless forcibly drawn back when there is a great lack of blood in the main channels, or driven by a fit of passion, when it flows to the greater circulatory vessels. As the blood of the veins naturally ascends incessantly or returns to the heart, so the blood of the arteries descends or departs from the heart; still if the smaller veins of the arms and legs be empty, the blood, filling the empty channels in succession, may descend in the veins. You perceive how the circulation is effected without any perturbation or confusion of fluids and the destruction of the ancient system of medicine."

Harvy's reply to the criticisms of Riolanus is moderate and dignified when it is remembered that some of the criticisms to which he had been subjected were personal and abusive. He began his reply to Riolanus in the following way, which is his only answer to the mass of his opponents: "It is now many years, most learned Riolanus, since, with the aid of the press, I published a portion of my work. But scarce a day, scarce an hour, has passed since the birthday of the Circulation of the Blood that I have not heard something for good or for evil said of this, my discovery. Some abuse it as a feeble infant, as yet unworthy to have seen the light; others, again, think the bantling deserves to be cherished and cared for; these oppose it with much ado, those patronize it with much commendation; one party holds that I have completely demonstrated the circulation of the blood by experiment, observation, and ocular inspection against all force and array of argument; another thinks it yet scarcely sufficiently illustrated—not yet cleared of all objections. There are some, too, who say that I have shown a vainglorious love of vivisections, and who scoff at and deride the introduction of frogs and serpents, flies, and others of the lower animals upon the scene as a piece of puerile levity, not even refraining from opprobrious epithets.

"To return evil speaking with evil speaking, however, I hold to be unworthry in a philosopher and searcher after truth; I believe that I shall do better and more advisedly if I meet so many indications of ill-breeding with the light of faithful and conclusive observation. It cannot be helped that dogs bark and vomit their foul stomachs, or that cynics should be numbered among the philosophers; but care can be taken that they do not bite and inoculate their bad humors, or with their dog's teeth gnaw the bones and foundations of truth."

NOTE.—I have been greatly helped in the preparation of this paper by the excellent translation of Harvey's works by Robert Willis, published by the Sydenham Society, 1847.—*Penn. Medical Journal.*

---

### NOTES ON THE PLAGUE IN CHINA AND INDIA.

By JOSEPH MARSHALL FLINT, of the Johns Hopkins University.

The June *Bulletin* of the Johns Hopkins Hospital contains a very instructive illustrated paper under above heading, from which we quote as follows:

The native and European quarters of the city of Hong-Kong present a strange contrast; for the European districts, from a sanitary point of view, are probably unexcelled in any city in the Orient, but the native quarter, notwithstanding some broad, fairly clean streets and new buildings, is really a whitened sepulchre. Here, in the tenements and side alleys, the coolies live in indescribable filth, the segregation and overcrowding being so great that at night the overflow sleeps in the streets. Many of these in the native districts are so narrow that one walking with outstretched arms can almost touch the buildings on opposite sides of the road. The houses are three or four stories high, and originally contained fairly large-sized rooms. The Chinaman, however, with his naturally frugal mind, subdivides them by cheap wooden partitions, and makes four rooms for one. With the decrease in the size of the rooms goes an increase in the number of their occupants, so that in one poorly ventilated tenement from thirty to forty natives are huddled together with less than 150 cubic feet of air-space per capita. As yet the coolie has not learned even the rudiments of personal hygiene, and the Chinese enjoy the unenviable distinction of being one of the filthiest people on the face of the earth. Apropos of this trait, some one has fitly called them practical Malthusians. One day in Hong-Kong we counted fourteen coolies pulling and pushing a meat-cart that could have been drawn easily by a single horse; so, in a country where a man and a horse show in a commercial ratio of fourteen to one, little in the way of civilization or personal esthetics can be expected. As a rule the common coolie never cleans either himself or his houses.

In speaking of these questions Lowson* says: "At the beginning of the outbreak a majority of the houses were in filthy condition. When to a mixture of dust, old rags, ashes, broken crockery, moist surface soil, is added fecal matter, and the decomposing urine of animals and human beings, a terribly unsanitary condition of affairs prevails; and that this is no overdrawn picture of what was to be met with in Tai-ping-shan many Europeans now know to their cost. One must recall, moreover, that Hong-Kong is Europeanized China, and that the conditions prevailing there do not compare with those found in Canton, for example—a typical Chinese city like those in other parts of the Celestial Empire. Indeed, comparing it with Canton there is something almost Utopic about the sanitary condition of the native quarters of Hong-Kong.

Once begun, the epidemic was fought by the following general sanitary measures: (1) Removal of the sick and dead. (2) Temporary segregation of those exposed while the premises were being disinfected. (3) Cleansing and disinfecting of "infected premises." (4) Disinfection of clothing. (5) General cleansing and limewashing of all tenement houses. (6) House-to-house visitation. (7) Disinfection of public latrines. These measures were all carried out with a considerable degree of success—much more success than attended the similar ones established later in India. This is due partly to the fact that the Chinaman is more easily bullied by the sight of power than the Hindu, and partly because the customs offended by the plague regulations are, in China, for the most part merely personal and are neither national nor religious.

During the height of the epidemic the medical staff had more to do than it could accomplish, but by the aid of a number of British soldiers, especially assigned to plague duty, managed to keep up

* Lowson: J. The Epidemic of Bubonic Plague in 1894. Medical Report, Hong Kong, 1895.

the routine work necessitated by the sanitary plague regulations. In this work the greatest opposition came from the unwillingness of the Chinese to send patients into hospitals and the resistance they made to house-to-house inspection. In the secretion of cases, moreover, they went to unheard-of extremes, and the district inspectors in their search for patients often saw sights that it seems almost impossible to believe. Dr. Lawson says, for example: "To overpaint the pitiable surroundings associated with plague work at the commencement of the epidemic would be impossible. I have entered a long, low cellar without any window opening, and with air entering only by a square open shaft from the level of the roof three or four stories high. Down one side of the shaft ran a broken earthware drain-pipe leaking freely, the contents streaming down the wall of the air-shaft to a shallow pool of filth which crossed the undrained floor of earth. Although it was broad daylight outside, a lantern was necessary to see one's way. On a miserable sodden matting, soaked with abominations, there were four forms stretched out. One was dead, the tongue black and protruding. The next had the muscular twitchings and the semi-comatose condition heralding dissolution. In searching for a bubo we found a huge mass of glands extending from Poupart's ligament to the knee-joint. This patient was beyond the stage of wild delirium. Sordes covered the teeth and were visible between the blackened and parted lips. Another sufferer, a female child, about ten years old, lay in the accumulated filth of apparently two or three days, unable to speak owing to the presence of enlarged cervical glands. The fourth was wildly delirious and was constantly vomiting. The attendant, the grandmother of the child, had a temperature of 103 degrees F., and could only crawl from one end of the cellar to the other. She was wet through and was herself doomed. This is no fancy sketch, but a true picture of how we found some of the patients at the outbreak of the scourge in Hong-Kong. No one unfamiliar with the horrors of the coolie accommodations in China could credit how the poor live in Hong-Kong, or could imagine how the horrors of their everyday life were intensified by the plague." In disinfecting some infecteded premises one day Dr. Lowson told us that in one room the inspectors were forced to dig through two feet of dirt and human excreta to reach the floor.

The mortality from plague in Hong-Kong among the Chinese varies between 91 and 93 per cent., so that almost all of those attacked, no matter what their treatment, die.

Between the plague in China and India there are many points of difference which depend, it seems, partly on the character of the natives. In China the pest is more fatal than it is in India, the death-rate among the Chinese being 93 per cent. and only about 82 per cent. among the low-caste Hindus, who are the heaviest sufferers from the disease in India. The general standards of life and personal hygiene are much lower among the Chinese than they are among the Hindus; but for some reason the epidemics are so much greater in India that the terrible effects of the disease are more obvious and its many horrors are impressed on the observer by the magnitude of the sufferings of the natives. Since the first outbreak in India, in 1896-7, the death-rate has constantly increased each year, until, in 1899, more than 50,000 people died of plague in the City of Bombay alone. Moreover, the disease has now spread in a large part of India and has appeared in Bengal, Madras and many points in the Bombay Presidency. The really serious part of the question is that apparently it is still on the increase, and precisely what the end will be no one at present can foretell.—*Carolina Medical Journal.*

---

OVEREATING.

Half the people we know have violent attacks of indigestion because they will persist in eating hearty meals when in an exhausted condition. They seem never willing or able to realize that there are times when the system is in no fit state to grapple with a full meal. They come in tired and hungry, almost ravenous, not thinking that maybe a good deal of what they consider hunger is gastric irritation, then sit down to a table covered with the substantials of life and deliberately go to work and overtax the already overstrained vital powers. No person should ever eat heartily when very tired. The wisest thing to do is to drink a cup of hot water with three teaspoonfuls of milk in it, sit down for five minutes, and then begin slowly to eat, masticating thoroughly. In a little while the vigor of the stomach will come back, and all will be well, one case of dyspepsia where now there are a dozen. It seems to be the most difficult of all things to properly control the appetite. It seems to be the master. It requires will-power to get it under control. When once mastered, something important has been accomplished in self-discipline.—*Exchange.*

